# Patient-reported outcomes and disease activity in giant cell arteritis: a longitudinal registry-based study

**DOI:** 10.1093/rheumatology/keag233

**Published:** 2026-05-05

**Authors:** Hans Kristian Skaug, Bjørg-Tilde Svanes Fevang, Jörg Aßmus, Andreas P Diamantopoulos, Geirmund Myklebust, Lene Kristin Brekke

**Affiliations:** Department of Rheumatology, Haugesund Hospital for Rheumatic Diseases, Haugesund, Norway; Department of Clinical Science (K2), Faculty of Medicine, University of Bergen, Bergen, Norway; Bergen Group of Epidemiology and Biomarkers in Rheumatic Disease (BEaBIRD), Department of Rheumatology, Haukeland University Hospital, Bergen, Norway; Department of Clinical Science (K2), Faculty of Medicine, University of Bergen, Bergen, Norway; Bergen Group of Epidemiology and Biomarkers in Rheumatic Disease (BEaBIRD), Department of Rheumatology, Haukeland University Hospital, Bergen, Norway; Centre for Clinical Research, Haukeland University Hospital, Bergen, Norway; Division of Internal Medicine, Department of Infectious Diseases, Akershus University Hospital, Lørenskog, Norway; Research Department, Hospital of Southern Norway, Kristiansand, Norway; Department of Rheumatology, Haugesund Hospital for Rheumatic Diseases, Haugesund, Norway; Bergen Group of Epidemiology and Biomarkers in Rheumatic Disease (BEaBIRD), Department of Rheumatology, Haukeland University Hospital, Bergen, Norway

**Keywords:** large vessel vasculitis, giant cell arteritis, disease activity, patient-reported outcomes, registry-based study, health-related quality of life, real-world data

## Abstract

**Objectives:**

The objective of this registry-based cohort study was to evaluate longitudinal associations between disease activity measures and patient-reported outcomes (PROs) in GCA, and to assess whether specific PRO domains reflect clinically active disease.

**Methods:**

Among all GCA patients registered in NorVas up to 12 December 2024, we selected patients who: (i) fulfilled the ACR 1990 classification criteria for GCA, (ii) had two PROs recorded at least once, and (iii) were included in NorVas at the time of diagnosis. HRQoL was assessed by RAND-12, using the physical (PCS) and mental (MCS) composite scores as outcomes. Visual analogue scales were used to assess pain, fatigue and global disease assessment. The association between the PROs and disease activity were evaluated using linear mixed effects models. We assessed the PROs over time and the difference in PROs between active and inactive disease.

**Results:**

We included 256 patients in the study with a median of three observations each, and a total of 1003 observations. All examined PROs showed a significant difference between active and inactive disease at baseline. Statistically and clinically significant differences were retained during follow-up for RAND-12-PCS (11.19 [95% CI: 5.67, 16.71]), pain (−12.64 [95% CI: −18.58, −6.70]) and global assessment (−9.92 [95% CI: −15.48, −4.35]).

**Conclusion:**

Our study demonstrates a consistent association between PROs and disease activity in GCA, most pronounced for the physical component of HRQoL, pain and global assessment. Patients with active disease showed statistically and clinically significant differences in PRO scores compared with those in remission, both at baseline and throughout follow‑up. While no single PRO domain can replace formal disease activity assessment, patterns across pain, fatigue and patient global measures may signal active disease and warrant clinical reassessment. Taken together, these findings indicate that selected combinations of PROs may serve as a useful adjunct in the monitoring of GCA.

Rheumatology key messages

- Patient-reported outcomes (PROs) are associated with disease activity in giant cell arteritis (GCA)
- PRO scores differ between active and inactive disease both at disease onset and during follow-up
- Our study can indicate a potential utilization of PROs in the monitoring of GCA

## Introduction

Giant cell arteritis (GCA) is a systemic vasculitis involving large and medium-sized arteries. It can present with various symptoms, ranging from irreversible vision loss to non-specific symptoms such as musculoskeletal pain, reduced mobility, fatigue or other constitutional complaints [1, 2]. As such, it is a disease that has the potential to influence patients’ health-related quality of life (HRQoL). Only a few studies have assessed HRQoL in GCA patients, and these studies differ regarding methodology and study design [3–6].

HRQoL and other patient-reported outcomes (PROs) capture the patients’ perception of symptoms, functioning and well-being. These aspects are not fully reflected by clinical examination or biomarkers. In GCA, disease activity indices primarily reflect inflammatory status, whereas PROs reflect disease impact. These complementary dimensions may align or diverge across the disease course and with treatment. Clarifying their association is therefore important for clinical interpretation of PROs and for patient‑centred monitoring in GCA. The relationship between disease activity and PROs has also been prioritized in the EULAR research agenda [7].

PROs are used to assess various aspects of the patients’ perceived health status using instruments designed to quantify these aspects, i.e. patient-reported outcome measures (PROMs). PROMs can range from comprehensive questionnaires composed of multiple items to simple tools, such as a single visual analogue scale (VAS). PROMs can be generic, i.e. suitable for application across different diseases or states of health, or disease-specific and tailored to a distinct disease or health status. HRQoL, pain and fatigue are all aspects that have been shown to be important for GCA patients [8, 9]. Although, less used in GCA, the patient’s global assessment of disease activity is a well-established outcome in rheumatology, particularly for rheumatoid arthritis [10].

The Norwegian Vasculitis Registry (NorVas) is a nationwide quality registry that aims to include all Norwegian patients diagnosed with systemic vasculitis. The registry was established in 2014 and gained status as a nationwide quality registry in 2016. By the end of 2024, NorVas received data from 17 out of 19 Norwegian rheumatology departments, resulting in a registry completeness of 60% [11].

In this registry‑based observational study, we examined associations between disease activity and routinely collected clinically relevant PROs in GCA, namely HRQoL, pain, fatigue and patient global assessment.

## Methods

### Patient selection

This registry-based study is conducted using routinely collected ‘real-world’ longitudinal data from NorVas. The study investigates GCA patients registered in NorVas up to and including 12 December 2024. Newly diagnosed patients were eligible for inclusion in the study if they met the 1990 American College of Rheumatology (ACR) Classification Criteria for GCA, as recorded in the registry, and had at least one clinical contact with a minimum of two PROs documented. Included PROs were the RAND-12 physical or mental composite scores (RAND-12-PCS or RAND-12-MCS), pain, fatigue and patient global assessment of disease activity. To ensure temporal alignment between diagnosis and registry inclusion, only patients whose inclusion date fell within 2 days of the documented date of diagnosis were retained for analysis. The registered date of treatment initiation was used to account for glucocorticoid treatment beginning before the first recorded PRO, but no inclusion or exclusion criteria were based on treatment exposure or glucocorticoid dose. For all included patients, we extracted data from all contacts with at least two PROs recorded. Thus, each individual patient could contribute multiple observations to our study. The risk of selection bias inherent to observational studies was assessed by comparing the population characteristics of the study sample with other cohorts of GCA patients and with the patients enrolled in NorVas but not included in our study. We assumed that the PROs were intercorrelated and treated them accordingly in our analyses.

### Patient reported outcomes

The NorVas registry collects HRQoL data from patients in connection with rheumatology consultations, measured by RAND-12 (also known as Veterans RAND 12 or VR-12). RAND-12 is a widely applied generic PROM derived from the longer RAND-36 [12]. The RAND-12 and RAND-36 are comparable to the first version of the generic questionnaires SF-12 and SF-36 [13]. The 12 items in RAND-12 can be used to make composite scores for the physical (PCS) and mental (MCS) domains of HRQoL [13]. PCS and MCS range from 0 to 100, with higher scores indicating desired outcomes. The Norwegian translation of RAND-12 has been validated and shown reliable in a Norwegian population [14, 15]. The questions included in RAND-12 can be found on the NorVas website [16, 17].

The registry also collects PROs regarding pain and fatigue, as well as the patient’s global assessment of disease activity. For these parameters VAS scores are collected, ranging from 0 to 100, with lower scores indicating the desired outcomes. The questions forming the basis for VAS-registration are: ‘How much pain have you had during the past week?’, ‘To what extent has feeling unusually tired or exhausted been a problem for you in the past week?’ and ‘We kindly ask you to assess the activity of your rheumatic disease during the last week. Taking all the symptoms into account, how do you think the condition is?’. A complete overview of the collected PROs is available on the NorVas website [18, 19].

The selection of PRO domains was pre-specified *a priori* and included HRQoL (PCS and MCS), pain, fatigue and patient global assessment. For interpretative clarity, PCS and MCS were considered key outcomes, whereas the remaining domains were treated as secondary or exploratory.

### Disease activity

At each NorVas-registered contact the physician records Kerr’s criteria, a global disease assessment, and level of C-reactive protein (CRP) as measures of disease activity. Erythrocyte sedimentation rate (ESR) is only recorded categorically as elevated or not elevated, as part of Kerr’s criteria.

Kerr’s criteria, originally developed for Takayasu arteritis, define disease activity according to the following four elements: (i) systemic features, such as fever or musculoskeletal pain, without any other explanation; (ii) elevated ESR; (iii) clinical symptoms of ischaemia, e.g. headache, visual disturbances, jaw or limb claudication, likely attributed to vasculitis; and (iv) typical angiographic features. One point is given for each element, and the disease is defined as ‘active’ if the Kerr score is >1 points [20].

The NorVas physician’s global disease assessment categorizes the disease activity as one of ‘onset’, ‘relapse’ (Kerr’s criteria > 1) or ‘remission’ (Kerr’s criteria ≤ 1). In our study, we needed to define disease activity as either ‘active’ or ‘inactive’. For this we used the physician’s global disease assessment together with CRP and Kerr’s criteria.

For each observation with a non-elevated ESR, we added one point to the Kerr’s criteria score if CRP was >10 mg/l, in accordance with the 2022 ACR/EULAR Classification criteria for GCA [21]. After this we re-evaluated the Kerr’s criteria score, and disease activity was defined as ‘active’ if the score was >1. For observations without documented Kerr’s criteria and CRP ≤10 mg/l, disease activity was defined as ‘inactive’.

Finally, for all observations, we compared the physician’s global disease assessment with the redefined disease activity. If disease activity, according to our definition, was ‘active’ while the physician’s global disease assessment was ‘remission’, the observation was excluded. Likewise, if our definition indicated ‘inactive’ disease while the physician’s assessment was ‘relapse’, the observation was also excluded. Eleven observations were excluded on these grounds.

### Statistical analyses

Descriptive methods were used to characterize the sample. We used linear mixed effects models with PRO depending on time, disease activity and their interaction adjusted for individual random intercept. We categorized time into ‘Inclusion’ and ‘Follow-up’. Adjustment variables were age, sex and treatment prior to inclusion, the latter defined as a dichotomous variable (yes/no). Additional models explored interactions between time and each adjustment variable. Model comparisons were made by estimating and plotting marginal means.

Missing data were handled using multiple imputation under the assumption of missing at random, with predictive mean matching as the imputation method [22]. The imputation model included the PROs, disease activity, age, sex, CRP, time from inclusion to observation, time from inclusion to treatment initiation, and individual identifier. Due to the limited number of observations per individual, most variables were imputed as single-level variables, except for time from inclusion to treatment initiation, which was specified as a level-2 variable. A total of 100 imputed datasets were generated (*m* = 100) with a maximum of 40 iterations per dataset (maxit = 40).

The chi squared test and Fisher’s test were used to evaluate associations between treatment prior to inclusion and disease activity at inclusion and at the first registered follow-up. We assessed correlation between the different PROs by scatterplots and Pearson’s correlation coefficient. *P*-values <0.05 were considered statistically significant. All data handling, analyses, and generation of figures and tables were performed in R v.4.5 (R Foundation for Statistical Computing, Vienna, Austria), using the package mice v.3.18.0 for multiple imputation [23–26].

### Ethical considerations

NorVas included patients only after informed consent up to and including 12 December 2024. This study was approved by the NorVas advisory board (reference number 2022/01) and by the Norwegian Regional Committees for Medical and Health Research Ethics (REK, reference number 264780).

## Results

**Figure 1:**
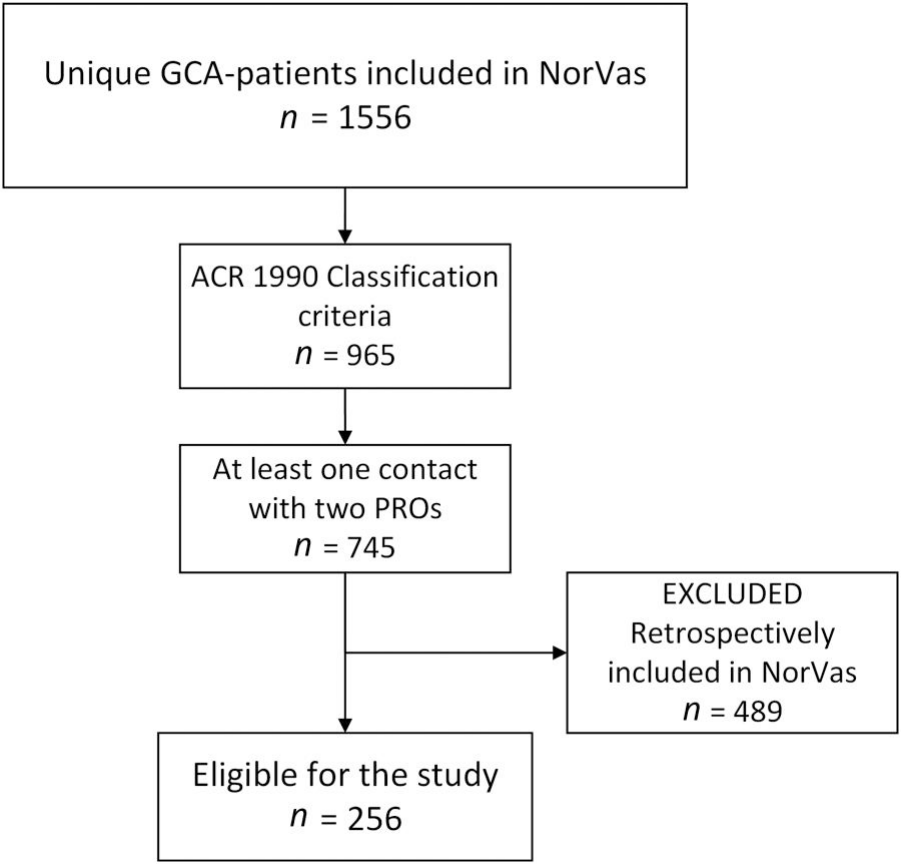
Flowchart depicting patient selection. GCA: giant cell arteritis; NorVas: Norwegian Vasculitis Registry; PRO: patient-reported outcome

We included 256 patients, of whom 160 (63%) were female (Fig. 1, Table 1). Each patient had a median of three observations, resulting in a total of 1003 observations. Follow-up data were available for 186 patients (73%).

**Table 1:** Population characteristics.

Characteristic	All patients (*n* = 256)
Age, median (IQR), years	72 (66, 78)
Female sex, *n* (%)	160 (63)
Active disease at time of inclusion, *n* (%) (*n* = 246)^a^	180 (73)
Active disease during follow-up, *n* (%)	28 (11)
Number of observations per patient, median (IQR)	3 (1, 5)
Number of observations with active disease, median (IQR)	1 (0, 1)
Medication started before time of inclusion, *n* (%) (*n* = 217)^a^	106 (49)
Time to first registered follow-up after inclusion, median (IQR), days	100 (49, 231)
Time to second registered follow-up after inclusion, median (IQR), days	232 (133, 447)
Patient-reported outcomes at time of inclusion	
Pain (VAS, 0–100), median (IQR) (*n* = 253)^a^	34 (8, 60)
Fatigue (VAS, 0–100), median (IQR) (*n* = 255)^a^	40 (11, 70)
Global assessment of disease activity (VAS, 0–100), median (IQR) (*n* = 252)^a^	40 (13, 67)
RAND-12-PCS (0–100), median (IQR) (*n* = 194)^a^	41 (25, 69)
RAND-12-MCS (0–100), median (IQR) (*n* = 191)^a^	56 (36, 75)

a PROs, information about medical treatment, and assessment of disease activity were not available for all patients; estimates are based on the available data. Regarding PROs, a higher score for pain, fatigue and global disease assessment indicates worse outcome, while for RAND-12-PCS and RAND-12-MCS a higher score indicates higher health related quality of life (HRQoL). IQR: interquartile range; MCS: mental composite score; PCS: physical composite score; PRO: patient-reported outcome; VAS: visual analogue scale.

At the time of inclusion 180 patients were defined as having active disease. Initiation of pharmacological treatment prior to inclusion was associated with disease activity status at time of inclusion (*P *< 0.001). Among 111 patients who had not initiated treatment prior to inclusion, 92 (83%) had active disease at inclusion. In contrast, among 106 patients who did not initiate treatment prior to inclusion, only 60 (57%) had active disease at the time of inclusion. At the first registered follow-up there was no association between disease activity and treatment prior to inclusion (*P *= 0.77). Correlation analysis of PROs confirmed moderately strong intercorrelations, thereby supporting the assumption underlying our methodology (Supplementary Fig. S1).

**Figure 2:**
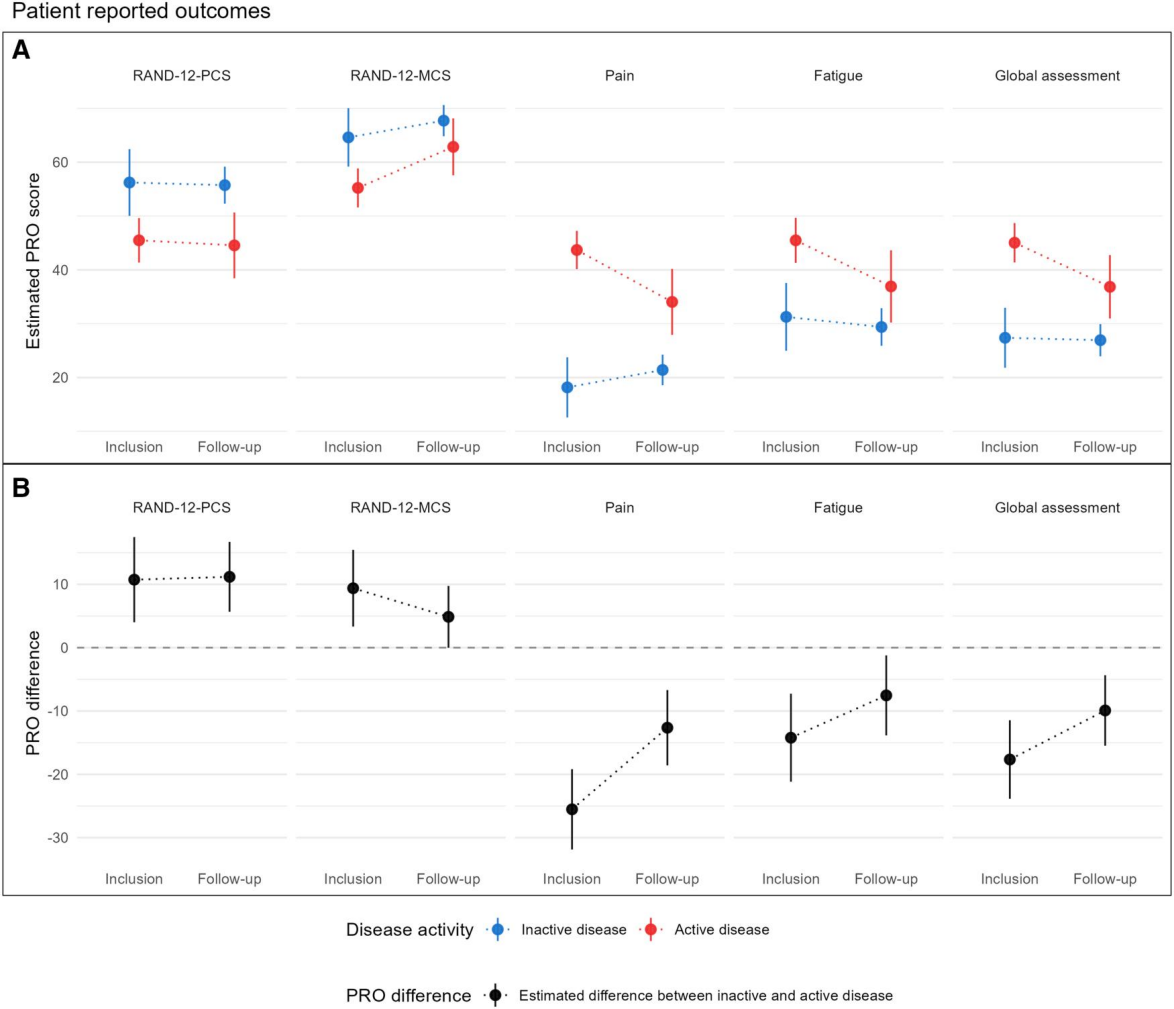
Estimated marginal means for each patient-reported outcome (PRO) by disease activity and time, categorized into ‘Inclusion’ and ‘Follow-up’. (**A**) Estimated PRO scores by disease activity. (**B**) Difference between PRO scores for inactive and active disease. Estimates are shown as points with vertical lines indicating 95% confidence intervals. The horizontal dashed line in (**B**) indicates zero difference. The linear mixed effects models were adjusted for age at diagnosis, sex and treatment before inclusion. The plotted estimates represent the average across these variables. For RAND-12-PCS and -MCS higher scores are better, and a positive difference indicates that PROs for inactive disease are higher, that is better, than for active disease. For pain, fatigue and patient-evaluated disease activity, lower score is better, and a negative difference indicates that PROs for inactive disease are lower, that is better, than for active disease. MCS: mental composite score; PCS: physical composite score

Most PROs improved over time (Fig. 2A and Supplementary Table S1). The selected model contained interactions between time and all the other variables, allowing the effects of both explanatory and adjustment variables to vary over time. The full model summaries are presented in Supplementary Data S1. Seemingly, the impact of disease activity on the PROs was larger at the time of inclusion compared with follow-up (Fig. 2B and Supplementary Table S1). Furthermore, the difference between PRO scores for inactive and active disease was significant for all PROs at both inclusion and follow-up, except for RAND-12-MCS at follow-up (*P *= 0.051). Statistically and clinically significant differences were retained during follow-up for RAND-12-PCS (11.19 [95% CI: 5.67, 16.71]), pain (95% CI: −12.64 [−18.58, −6.70]) and global assessment (−9.92 [95% CI: −15.48, −4.35]) (Fig. 2B and Supplementary Table S1).

## Discussion

To our knowledge, this is the first study to longitudinally assess the association between PROs and disease activity in a large, registry-based cohort of GCA patients. We found that PRO scores were significantly different between patients with active and inactive disease, both at inclusion and at follow-up. Only the difference in the mental part of the quality of life-questionnaire (RAND-12-MCS) did not reach statistical significance at follow-up, although the trend mirrored that of the other PROs. While it is expected that patients with active disease report lower HRQoL, the novelty of our study lies in demonstrating this association over time, using repeated measures and accounting for both between-patient and within-patient variation.

Previously, one longitudinal study has investigated HRQoL in patients with GCA based on data from the GiACTA trial, but that study did not evaluate the role of disease activity [6]. Strand *et al.* found that baseline HRQoL was low, and that during the study period there was a significant improvement, most evident for patients receiving tocilizumab [6]. Other studies addressing HRQoL in GCA have mostly been cross-sectional in design aiming to evaluate PROs for GCA patients with or without comparison with a reference population [3, 4].

The PROs used in this study are all generic instruments, not specifically designed for GCA. Nevertheless, they can still capture aspects relevant to maintaining HRQoL, including factors associated with GCA. The Outcome Measures in Rheumatology (OMERACT) Vasculitis Working Group has repeatedly highlighted the need for disease-specific PROMs for GCA [27, 28]. Recently, two disease-specific PROMs for GCA have been developed and validated [9, 29, 30]. However, the instrument published by El Miedany *et al*. [30] is not a pure PROM, as it incorporates clinical and laboratory components; it therefore represents a composite disease activity index rather than a patient-reported measure. Nonetheless, both instruments showed moderate correlation with HRQoL, suggesting a substantial thematic overlap. Additionally, pain and fatigue have been put forth as two important domains of quality of life regarding GCA [8, 9].

HRQoL is a complex construct to evaluate. Our study is based on data obtained using a well-established instrument for measuring HRQoL, as well as three visual analogue measures. When assessing differences in these scores, the minimal clinically important difference (MCID) must be considered after statistical significance is assessed. It is generally accepted that MCID for the three VAS scales is 10 mm [31]. The MCID for a deterioration in the physical and mental composite scores for SF-36 has been defined as −0.8 [31]. It may be reasonable to assume a similar MCID estimate for RAND-12, as the scales are closely related. As the estimated differences in our study account for both within and between individual variability, the MCID values are not directly transferable to the differences estimated by our models. Still, it is encouraging to see that several of the estimates lie well above the MCIDs.

Although our study does not share the controlled environment of a prospective clinical study, the NorVas registry is becoming a well-established infrastructure for data collection in most rheumatology departments throughout Norway. The registry’s level of completeness is steadily increasing, enhancing its potential as a resource for observational research and quality improvement [11]. The key characteristics of our study population are comparable to other published GCA cohorts although the proportion of female patients were somewhat lower than in some cohorts [2, 32–37].

A common workflow in the clinics is that patients register the PROs first, and the rheumatologists evaluate disease activity thereafter. As such, it is reasonable to assume that the registered PROs are not biased by the physician’s evaluation but rather reflect the patient’s true perceived health status. While it is possible that the patient’s PROs could influence the physician’s evaluation and introduce bias, we assume that the physician’s assessment is based on a comprehensive clinical picture, including signs and symptoms, laboratory findings, and imaging results, which mitigates this potential source of bias. Another strength of our study is the large sample size and the total number of observations. Limitations of our study include the low number of follow-up contacts per patient and variability in the timing between contacts. Additionally, we were unable to fully evaluate the effect of treatment and the potential impact of disease damage during follow-up. Lastly, missingness in the registry could potentially introduce bias into the study sample.

The monitoring of GCA patients remains an area with unresolved issues [7]. While it is widely accepted that close monitoring is essential during the early disease phase until stable remission is achieved, there is a lack of evidence to guide monitoring practices once remission is established and after pharmacological treatment has been discontinued. Consequently, monitoring strategies may vary considerably across clinical settings. A tool to support remote monitoring of patients in stable remission could reduce such variation by facilitating standardized follow-up practices, thereby potentially improving the quality and consistency of care.

In Norway, NorVas is integrating collection of PROs in the routine rheumatology care for GCA patients. However, as the clinical utility of these data remains unclear, PROs may go unnoticed by clinicians. Consequently, the patients may feel that their reported outcomes are not valued. Our findings may support a potential new role for PROs in clinical practice. We demonstrate that PROs can reflect changes in disease activity in GCA patients followed longitudinally, an effect that was most evident for the physical component of HRQoL, pain and the patient’s global assessment. As such, these PROs could, either by themselves or in combination, potentially serve as indicators for disease activity in GCA. Further, these indicators could form a basis for remote monitoring in the follow-up of GCA patients. This potential application warrants further investigation, ideally through prospective, controlled clinical studies.

## Conclusions

Our study demonstrates a consistent association between PROs and disease activity in GCA. The association was most pronounced for the physical component of HRQoL, pain and global assessment. Patients with active disease showed statistically and clinically significant differences in PRO scores compared with those in remission, both at baseline and throughout follow‑up. While no single PRO domain can replace formal disease activity assessment, patterns across pain, fatigue and patient global measures may signal active disease and warrant clinical reassessment. Taken together, these findings indicate that selected combinations of PROs may serve as a useful adjunct in the monitoring of GCA. However, further research is needed to better understand the associations and variability between PROs and disease activity over time.

## Supplementary Material

keag233_Supplementary_Data

## Data Availability

The patient data used in this study contain sensitive information and cannot be made publicly available. The complete data used in this study are provided by NorVas and are available upon approved application to the registry.
