# Supplementary material for: Patient-reported outcomes and disease activity in giant cell arteritis: a longitudinal registry-based study
Source: Rheumatology (Oxford). 2026 May 5;65(5):keag233. doi: 10.1093/rheumatology/keag233 (PMC13215876; doi:10.1093/rheumatology/keag233)
Supplement: keag233_Supplementary_Data [file keag233_supplementary_data.zip › rhe-25-2506-File005.docx]

**Supplementary Figure S1**


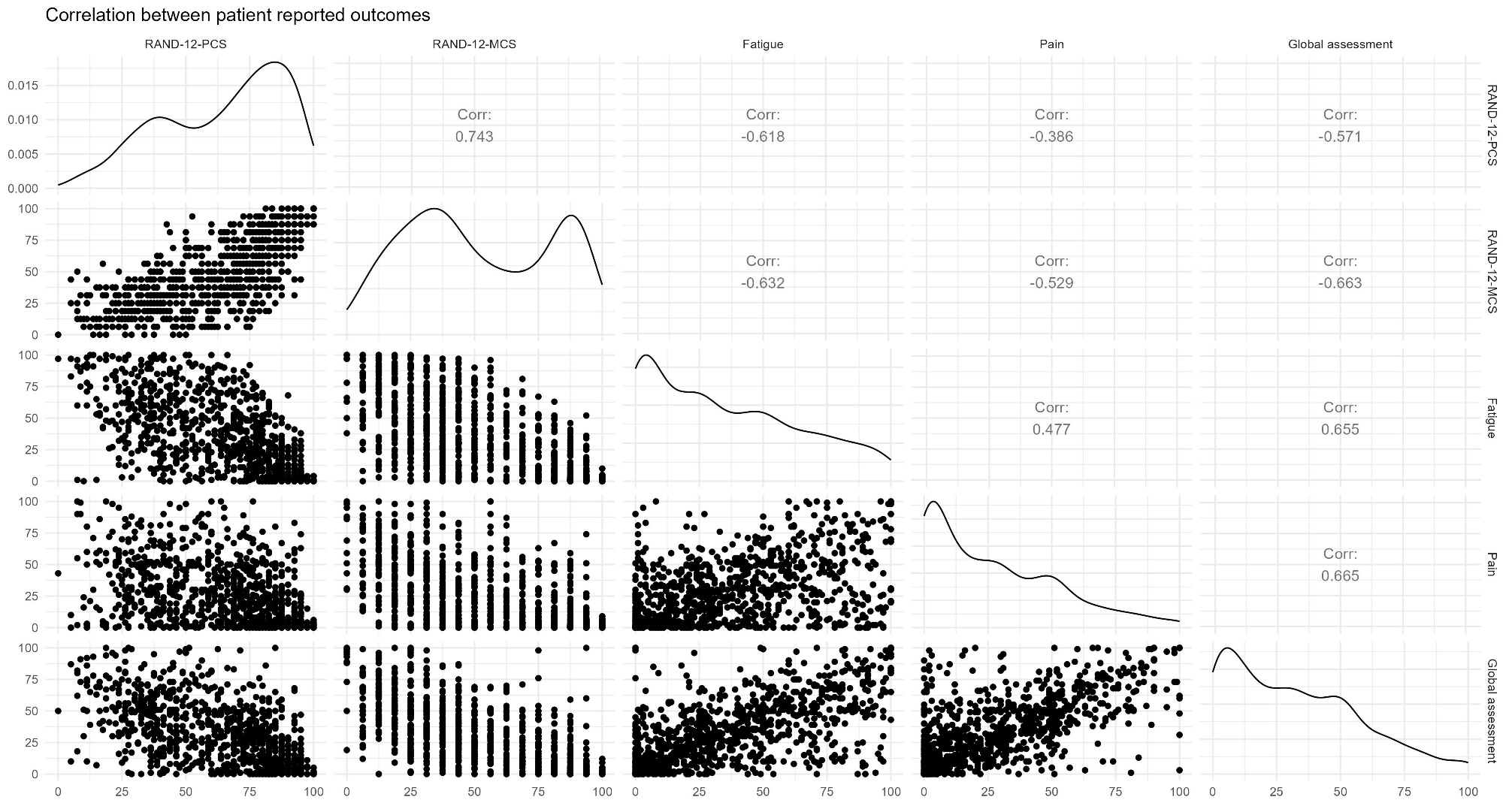


Plot showing the distribution of each patient reported outcome (PRO), a scatterplot comparing each pair of PROs and the corresponding correlation coefficients.

**Supplementary Table S1**

Estimated marginal means for each patient-reported outcome (PRO) by disease activity and time, categorized into “Inclusion” and “Follow-up”.

The linear mixed effects models were adjusted for age at diagnosis, sex, and treatment before inclusion. The estimates represent the average across these variables. For RAND-12-PCS and -MCS higher scores are better, and a positive difference indicates that PROs for inactive disease are higher, i.e. better, than for active disease. For pain, fatigue, and patient evaluated disease activity lower score is better, and a negative difference indicates that PROs for inactive disease are lower, i.e. better, than for active disease.
